# Critical uncertainties in preclinical research: Navigating trust, technology, and ethics

**DOI:** 10.1016/j.nsa.2026.107001

**Published:** 2026-04-10

**Authors:** Indrek Heinla, Vootele Voikar, Hilary Gates, Rasneer Sonia Bains, Sara Wells, Silvia Mandillo, Maša Čater, Kristi Kukk, Lars Lewejohann, Dragan Hrnčić, Daniela Duarte-Domingues, Philipp Villiger, Leonardo Restivo, Sabine M. Hölter

**Affiliations:** a University of Tartu, Institute of Psychology, Näituse 2, 50409, Tartu, Estonia; b Estonian Research Council, Department of Strategic Analysis, Soola 8, Tartu, 51004, Estonia; c Laboratory Animal Center, Helsinki Institute of Life Science, University of Helsinki, Finland; d Mary Lyon Centre @ MRC Harwell, Harwell Campus, Oxfordshire, OX11 0RD, UK; e Institute of Biochemistry and Cell Biology, CNR-National Research Council, Monterotondo, Rome, Italy; f University of Ljubljana, Biotechnical Faculty, Domžale, Slovenia; g German Centre for the Protection of Laboratory Animals (Bf3R), German Federal Institute for Risk Assessment (BfR), Berlin, 12277, Germany; h Institute of Animal Welfare, Animal Behavior and Laboratory Animal Science, School of Veterinary Medicine, Freie Universitat Berlin, Berlin, 14163, Germany; i Faculty of Medicine, University of Belgrade, Belgrade, Serbia; j Paris Brain Institute - ICM, Sorbonne Université, Inserm, CNRS, AP-HP Hôpital de la Pitié Salpêtrière, Paris, France; k Zurich Integrative Rodent Physiology ZIRP, Department of Physiology, Universität Zürich UZH, Zurich, Switzerland; l Department of Fundamental Neurosciences, Faculty of Biology & Medicine, University of Lausanne, Lausanne, Switzerland; m Institute of Experimental Genetics, Helmholtz Munich, German Research Centre for Environmental Health, Neuherberg, Germany; n German Center for Mental Health (DZPG), Partner Site Munich, Germany

**Keywords:** New approach methodologies, Animal research, Strategic foresight, Scenarios, Trust in science, Competitiveness

## Abstract

Animal use in preclinical research is facing growing scientific, ethical and political scrutiny in Europe. Researchers are increasingly required to justify in vivo studies and are urged to replace them with New Approach Methodologies (NAMs), even where these are not yet fit for purpose. In response to this pressure, members of the COST Action CA20135 “Improving biomedical research by automated behaviour monitoring in the animal home-cage” (TEATIME) held a Strategic Foresight Workshop in March 2025 to examine the future of animal research in Europe. The resulting scenarios reveal risks of over-regulation, outsourcing animal experiments to countries with weaker standards, and erosion of research quality, but also highlight opportunities to combine validated NAMs with high-quality in vivo work under robust ethical oversight. This short communication summarises the workshop's main insights and argues for evidence-based, transparent and internationally aligned regulation that protects both animal welfare and the scientific value of preclinical research.

## 1 Introduction: eroding trust in science

We live and work in a complex and unpredictable environment. Recent examples, such as the COVID-19 pandemic, climate-related disruptions, and (geo)political instability have demonstrated how quickly science can be affected by global crises, underscoring the fragility of research ecosystems. Volatility and ambiguity are not transient phenomena, nor do they leave the academic world untouched. On the contrary, politics and policy changes affect every aspect of scientific practice. To mention just a few examples of forthcoming policy changes, there is the UK Government Replacing Animals in Science Strategy (GOV UK Department for Science et al., 2025), the EU roadmap for phasing out animal testing for chemical safety assessment (Walder et al., 2025), and the FDA Modernization Act 2.0 in the US (Adashi et al., 2023). The systems that support scientific rigor and new discoveries are struggling to keep pace with those changes. The threat is not a single event or trend, but a convergence of critical vulnerabilities that, if left unaddressed, could undermine the credibility, effectiveness, and ethical foundation of biomedical science.

The care and use of animals for scientific purposes is governed by internationally established principles of Replacement, Reduction, and Refinement and the EU legislation on animals in science centers on the principle of the “Three Rs”, based on the seminal work of William Russell and Rex Burch (Russell et al., 1959). A narrow focus on replacement over the readiness of New Approach Methodologies (NAMs) to complement in vivo research has created situations where premature implementation risks compromising scientific quality.

Emerging NAM technologies such as organoids, *in vitro* and *in silico* models (including increasingly AI-based approaches) are often heralded as the future of biomedical research (Harrison, 2025; Herron et al., 2025). While their potential is undeniable, the reality is more complex. NAMs are already proving highly effective in defined, limited contexts, such as high-throughput toxicology screening, organ-on-chip models for barrier tissues, and computational pharmacokinetics, but recognizing both their strengths and limitations is essential to avoid hype-driven misapplications. These tools are not yet capable of fully replacing animal models as so-called “alternative technologies” to animal research, especially in areas involving complex physiological interactions, immune responses or behavioural studies (Lloyd, 2025; Reardon, 2025). Overreliance on unproven alternatives can cause both scientific setbacks and ethical backlash. If overpromising and underperforming technologies continue to dominate the narrative, they may undermine public trust, stall regulatory acceptance, and divert funding from more validated and impactful research avenues.

Another pressing uncertainty is whether society continues to perceive science - especially preclinical research - as trustworthy. Verified scientific knowledge is competing with influencer rhetoric and AI-based opinions which are often the result of biased prompting. Misinformation, ethical concerns about animal testing, and a lack of transparency and insufficient public communication about scientific results obtained from animal studies, have created a credibility gap (Miller et al., 2025). Surveys (European_Commission, 2010; Ipsos) reveal that while a majority of EU citizens support animal research, when linked to medical benefit, concerns about transparency and necessity remain widespread, highlighting the need for open communication. Another example is Switzerland, where recently the Swiss population voted on a proposal to completely ban animal experimentation and the import of products developed through animal testing. Voters rejected the initiative by 80%. However, the issue persists, as new proposals to ban animal experimentation keep emerging.

If that pattern continues and public trust deteriorates further, it could lead to stricter regulations and reduced funding driven by public pressure rather than scientific rationale, and a growing disconnection between researchers and the communities they aim to serve. To be clear, we stand for the **meaningful and responsible use of animals**, with replacement applied wherever scientifically possible, but without compromising the gain in knowledge. Transparency in communicating how and why animals are used is a cornerstone for building this balance and ensuring that public concerns are addressed without eroding scientific quality (for example see (Understanding_Animal_Research; EARA)).

## 2 Main part: foresight as a framework to find a solution

**Fig. 1:**
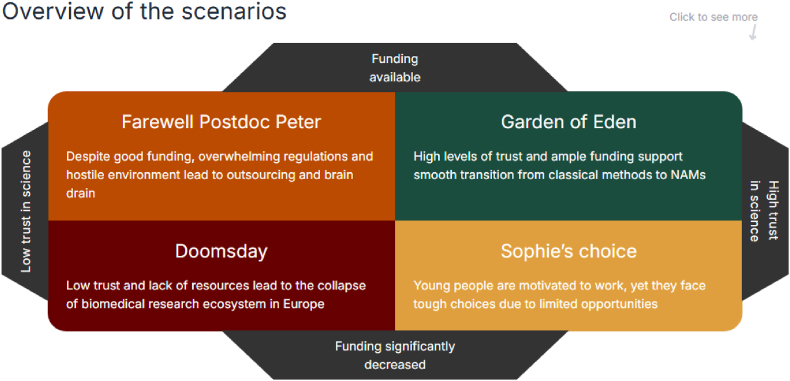
The four scenario quadrants emerging from the TEATIME foresight workshop on the future of preclinical research. The scenarios were developed after identifying two key uncertainties: the level of societal trust in science and availability of funding. Each quadrant represents a distinct potential future, that can be used to identify the strategic implications for researchers, policymakers, and institutions. Figure designed by science reporter Jaan-Juhan Oidermaa. This is the link to the interactive figure: https://novaator.github.io/Graafik/Heinla.html.

To systematically identify challenges such as those outlined above and to explore any others potentially on the horizon, the TEATIME consortium^1^ organized a strategic foresight workshop for its members. Strategic foresight combines structured horizon scanning (to systematically identify emerging trends, risks, and signals of change), scenario building (to construct plausible future contexts to explore potential outcomes), and backcasting exercises (starting from a desired future outcome and working backward to identify the actions and decisions needed to achieve it). Together these can help researchers anticipate disruptive changes and prepare adaptive strategies (Chermack, 2005; Commission et al., 2023, 2025; Meissner et al., 2013). By applying these principles, we developed plausible future scenarios (Fig. 1) to explore how combinations of different critical uncertainties (i.e variables exhibiting high impact and high uncertainty) might shape the future of research ecosystems and identify actionable steps we can take today to prepare for inevitable changes.

The most critical and impactful uncertainties in biomedical research we identified were:

(a) Whether science in general - and preclinical research in particular - will be perceived as trustworthy (both reliable and ethical) by society,
(b) The amount of funding available.

One such scenario, created during the workshop was titled “Farewell Postdoc Peter”. It focused on the issues of Trust in Science. The scenario's title symbolized the growing exodus of young researchers (like “Peter”) from academic careers, driven by systemic pressures and lack of societal trust in science. It offers a sobering look at what might happen when technological progress outpaces societal acceptance and regulatory alignment, depicting a world where technology and financial resources are available; however, the societal acceptance and understanding is dwindling.

The main **losers** in this scenario are laboratory animals. The EU is currently one of the most virtuous political entities in terms of regulations on the use of animals in research with high ethical standards and strict oversight. Overregulating animal research, as some advocacy groups currently propose both in the EU and in the US, would not eliminate the practice - it would merely shift it to countries with lower animal welfare standards (Mertz et al., 2024). Patients are also among the losers in this scenario, as breakthroughs in preclinical science could fail to translate into clinical application (Lloyd, 2025; Reardon, 2025).

This scenario highlights a broader dilemma faced by many young scientists across Europe. While individual researchers seek environments where they can thrive, the structural issues within the system (such as precarious contracts, lack of career path and funding, etc.), point to deeper tensions. Several recent surveys (including Eurodoc's 2024 Postdoc Survey (Parada et al., 2025) and analyses by CESAER (CESAER, 2024)) indicate that up to one-third of early-career researchers in Europe consider moving abroad due to limited opportunities and structural barriers, contributing to an ongoing „brain drain“ towards countries with more flexible research frameworks. While technological tools and funding are available in Europe, their use is hindered by strict regulations, public distrust, and ideological pressure. This is particularly true for fundamental research, which is necessary for innovation, even though it might not have an immediate application. These arguments raise important questions, not just about personal career paths, but about Europe's position and competitiveness in the global research landscape and its long-term strategy.

**The winners** in this future are tech-driven startups and private labs that can maneuver around constraints and operate within more flexible regulatory frameworks. Also gaining ground are countries and regions with supportive political climates and **pragmatic regulation**, which attract talent and investment (Fieldhouse, 2025). Certain campaigning organisations and activist movements also increase their influence by shaping public opinion and steering policy - even when their positions are not always grounded in scientific evidence. Driving critical research mainly into companies will lead to a less transparent, privately controlled environment and, consequently reduces opportunities for open data sharing and reproducibility checks.

## 3 Conclusion: the way forward

The bigger question is not whether animal research should be allowed or prohibited altogether, but whether we choose to maintain and improve strict ethical oversight within EU institutions – or outsource these activities to settings or countries where ethical and scientific standards may be significantly lower. Once that shift towards outsourcing occurs and local expertise and infrastructure are lost, rebuilding them becomes an arduous, if not impossible, task.

We advocate for responsible use of animals, prioritizing alternatives whenever they are viable, since these are complementary approaches in biomedical research. Depending on the context of use, both approaches can provide human relevant mechanistic insight and can have a good alignment with clinical conditions. Achieving the right solution requires balancing different interests and priorities. Policymakers, but also scientists, need to play their part in this. For that, full implementation of existing guidelines for best practice in laboratory animal research (such as PREPARE (Smith et al., 2018), EQIPD (Bespalov et al., 2021), and ARRIVE (Percie du Sert et al., 2020)) and of best practice guidelines for ensuring reproducibility, validity and responsible reporting of *in vitro* research (such as RIVER (The_RIVER_working_group) and GIVIMP (OECD, 2018)) is necessary. Taken together, these frameworks converge on a common principle: research quality depends less on novel methods than on rigorous implementation, transparency, and accountability. These guides must be followed by researchers, but also, and possibly even more importantly, by funders and publishers, in order to ensure high-quality research. Moreover, NAMs are not going to be better methods unless researchers learn from previous shortcomings in *in vitro* research, such as inadequate reporting, poor model validation and selective data presentation and ensure that reproducibility and validity guidelines apply as addressed by the RIVER Recommendations (The_RIVER_working_group). In an article focusing on chemical safety assessment, existing opportunities and challenges for the wider exploitation of NAMs have recently been discussed in a broader context by Sewell et al. (2024).

To ensure that Europe remains competitive in biomedical innovation while safeguarding high ethical standards, policymakers should prioritise targeted investment in complementary technologies that are either already validated or promising new developments. At the same time, it is of importance to continue funding animal research where alternatives are not yet appropriate. In short, funders should support **the methods best suited to answer the scientific questions**, regardless of whether they involve animals or other models. This requires sustained funding streams that bridge the gap between promising early-stage innovations and their regulatory approval, ensuring that alternatives are not only developed but also trusted and adopted. Meanwhile, regulatory frameworks should become more agile, enabling controlled, small-scale testing of new methods without undue bureaucratic delays. International alignment of standards is equally crucial, since biomedical research and regulation are increasingly globalized. Europe's leadership role depends on collaborative, not insular, strategies. By coupling strategic investment with flexible, science-driven regulation, Europe can accelerate the adoption of effective alternatives, maintain research excellence, and avoid the unintended consequence of shifting critical preclinical work to jurisdictions with weaker oversight.

Are you a preclinical researcher? If so, you have a critical role to play in shaping the policy and public narrative around your work. Engage actively in professional associations, advisory boards, and public debates to ensure that both regulations and public opinion are grounded in scientific evidence. Evaluate emerging technologies - whether *in vitro* models, *in silico* (including AI-based tools), organoid systems or others - with a critical and evidence-based mindset, avoiding both hype and undue scepticism while prioritizing reproducible, validated solutions. Strengthen your international collaborations to navigate diverse regulatory landscapes, maintain access to cutting-edge methods, and ensure that promising research can move forward even when local conditions become restrictive. Ultimately, the future of preclinical research will be defined not only by the technologies we develop, but by the integrity, responsibility, and transparency with which we apply them.

## Author contributions

All authors contributed to the Strategic Foresight Workshop. The first draft of the manuscript was written by IH and SMH. All authors commented on the draft and read and approved the final manuscript.

## Funding

This article is based upon work from the COST Action ‘Improving biomedical research by automated behaviour monitoring in the animal home-cage’ (CA20135 TEATIME) supported by COST (European Cooperation in Science and Technology). SMH was supported in part by the German Center for Mental Health (DZPG) through grant 01EE2503E from the Federal Ministry of Research, Technology and Space (Bundesministerium für Forschung, Technologie und Raumfahrt [BMFTR]).

## Declaration of competing interest

The authors declare that they have no known competing financial interests or personal relationships that could have appeared to influence the work reported in this paper.

## Footnotes

1 The COST Action CA20135 („TEATIME“) is a network of dedicated European preclinical researchers committed to improving quality of preclinical research by using high throughput and low-stress methods in the animal home cage.
